# An Unknown Foreign laryngeal Object: an exotic complication of skull base osteoradionecrosis

**DOI:** 10.1007/s00405-024-08507-1

**Published:** 2024-02-21

**Authors:** Stefan Grasl, Christian Wassipaul, Gregor Fischer, Christoph Arnoldner, Stefan Janik

**Affiliations:** 1 Department of Otorhinolaryngology, Head and Neck Surgery, Medical University of Vienna, Vienna, Austria; 2 Department of Biomedical Imaging and Image-Guided Therapy, Medical University of Vienna, Vienna, Austria; 3 Department of Otolaryngology, Karl Landsteiner University Hospital, Krems, Austria

**Keywords:** Skull base, Osteoradionecrosis, Radioosteonecrosis, Airway emergency, Complication

## Abstract

**Background:**

Osteoradionecrosis (ORN) of the skull base is a rare complication after head and neck radiation with a broad variety of subsequent complications.

**Methods:**

A 68-year-old woman with a complex oncological history (right-sided sphenoid meningioma; left-sided neck metastasis of a Cancer of Unknown Primary—CUP) was admitted with a right-sided epi—/ oropharyngeal mass and severe pain exacerbations for further evaluation. CT scan revealed an advanced ORN of the skull base with subsequent abruption of the ventral part of the clivus. This dislocated part of the clivus wedged in the oropharynx for 48 h and then moved towards the larynx, resulting in dyspnea and almost complete airway obstruction.

**Results:**

Due to the dangerous airway situation, an urgent exploration and removal of the dislocated clivus was necessary. After a potential cervical spine instability was ruled out, the patient’s airway was initially secured with an awake tracheotomy and the clivus was removed transorally. The tracheostomy tube was removed during the ongoing inpatient stay, and the patient was discharged with significant pain relief.

**Conclusions:**

The present case illustrates an orphan complication of skull base ORN resulting in a major airway emergency situation.

## Introduction

Osteoradionecrosis (ORN) of the skull base is a rare but feared complication of head and neck radiation with potentially devastating outcome. First described in 1926 by Ewing, ORN implicates radiation-induced chronic inflammation with collagen and endothelial cell degradation that causes increased bone matrix absorption. Therefore, head and neck ORN manifests as exposure of bone without soft tissue coverage, severe necrosis and tissue defects [1–5]. The most significant contributing risk factors are the extent of the radiation field with radiation doses above 60 Gy, accelerated fractionation without dose reduction, radiochemotherapy, poor oral hygiene and nutritional status as well as any surgical trauma such as tooth extractions. [6–11]

The management of ORN may be either conservative or surgical. Conservative treatment options include the application of hyperbaric oxygen (HBO) therapy and/or a mixed broad-spectrum antibiotic therapy combined with pentoxifylline, clodronate and tocopherol (PENTOCLO scheme) [12–18]. The surgical treatment, in turn, requires the removal of necrotic, floating bone, known as sequestrectomy and the reconstruction with healthy soft tissue. This can be achieved with local, regional or free flaps in either an open or endoscopic approach. These treatment strategies have shown an overall success rate of around 75%. [12]

Until now, plentiful complications of skull base ORN have been reported, ranging from major vascular bleedings, pain, cranial nerve palsies, cerebrospinal fluid (CSF) leak, pneumocephalus, meningitis, brain abscess up to temporal lobe necrosis in more than 10% [19–22]. The present case depicts another unusual complication of skull base ORN causing a dangerous airway situation.

## Case report

A 68-year-old woman was admitted to the Department of Otorhinolaryngology, General Hospital of Vienna, Medical University of Vienna, with severe right-sided pain exacerbations to further evaluate a right-sided naso—/ oropharyngeal mass. Due to an oncological history of head and neck tumors, there was a strong suspicion of a new or recurring malignant disease.

### Medical history

Regarding her oncological history, in 2011 she was diagnosed with a right-sided sphenoid meningioma and a left-sided neck metastasis of a CUP in 2012. The meningioma (08 / 2011) initially had a volume of 23.6 cm^3^ and infiltrated the cavernous sinus also causing a right-sided abducens palsy. It was treated with gamma-knife surgery with14Gy at the border area and 35 Gy in the center with a 40% isodose line with eleven isocenters (collimators; 4 mm, *n* = 1; 8 mm, *n* = 3; 14 mm, *n* = 1; 18 mm, *n* = 6).

In May 2012, the patient noticed a suspicious neck lump in level V and therefore underwent a diagnostic lymph node sampling. This turned out to be a lymph node metastasis of an intermediate differentiated squamous cell carcinoma without a clinically apparent primary tumor (CUP). Therefore, she underwent a thorough CUP check-up and subsequently therapy consisting of panendoscopy, completion of left-sided neck dissection and adjuvant radiochemotherapy. Due to the reduced general condition, the patient received only two of five scheduled cycles of cisplatin in a reduced concentration of 30 mg/m^2^ weekly. According to former CUP treatment recommendations, pharynx, larynx and neck levels Ib to V were radiated on both sides with a dosage of 50 Gy, the left level V neck with a boost up to 60 Gy, and the right parapharyngeal space, floor of mouth, soft palate and the junction between oro- and nasopharynx received an additional boost up to 70.4 Gy (2 Gy single dosage). [23, 24]

Signs of ORN were first present in 2016 when the patient presented with maxillary and mandibular ORN.Therefore, she underwent several long-term antibiotic treatments as well as surgical debridements. A partial mandibulectomy was finally performed in 2019, and there have been no apparent signs of ORN since then.

### Clinical presentation and treatment

From a clinical point of view, the patient now presented with trismus, severe right-sided pain (Numeric Rating Scale / NRS 10/10), a solid lesion in the oropharynx/nasopharynx and complete blockage of the right choana. Due to her former oncological and ORN therapies, she was already entirely dependent on a gastrostomy tube. Further imaging studies were scheduled and sufficient pain management was established. The solid lesion was biopsied transorally and histologically proved to represent inflammatory material without evidence of malignant cells.

**Fig. 1:**
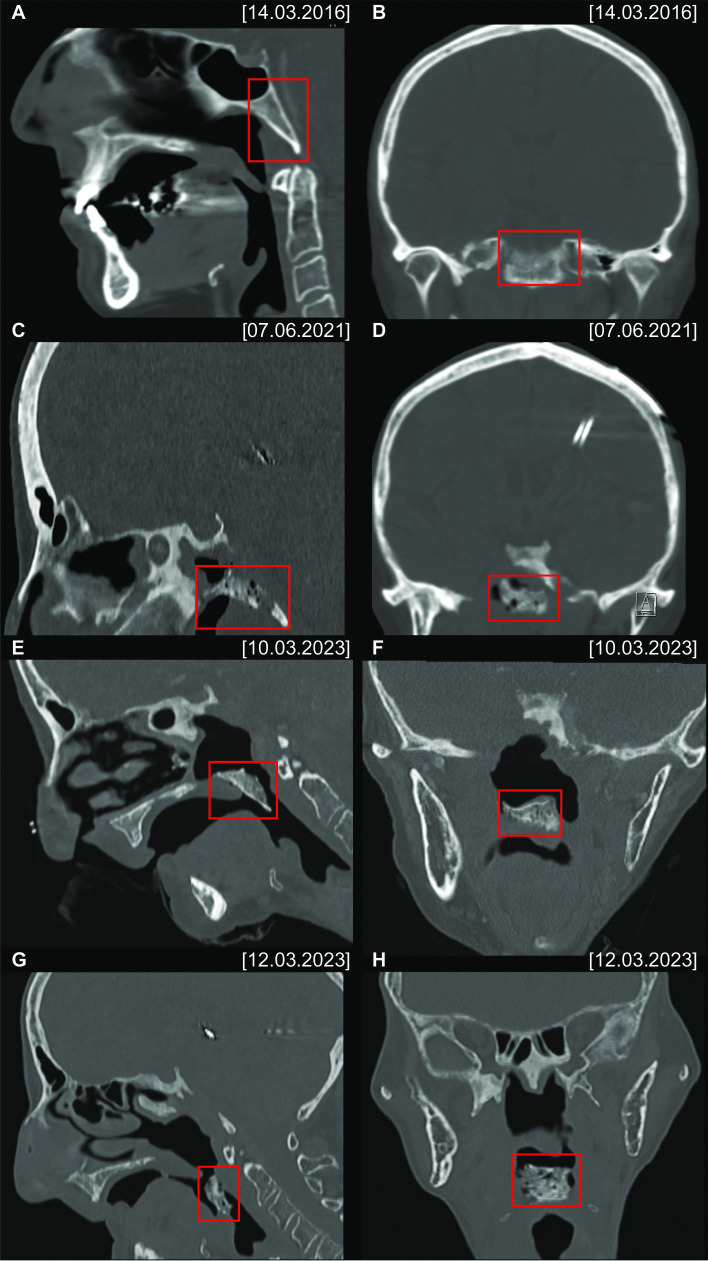
CT scans in the bone window (sagittal: **A**, **C**, **E**, **G**; coronal: **B**, **D**, **F**, **H**) show severe radio osteonecrosis of the anterior skull base with a chip fracture of the clivus (red rectangle) and the progredient dislocation

The subsequent head and neck CT scan revealed an advanced ORN of the skull base with subsequent abruption of the ventral part of the clivus with caudal dislocation towards the oropharynx (Figs. 1E, F and A). Soft tissue was present in the former location of the clivus and no pneumocephalus or CSF leakage had developed. Within the next 48 h, a further dislocation of the fractured clival sequestrum occurred, resulting in an almost complete obstruction of the laryngeal inlet with severe dyspnoea (Figs. 1G, H and B).

**Fig. 2:**
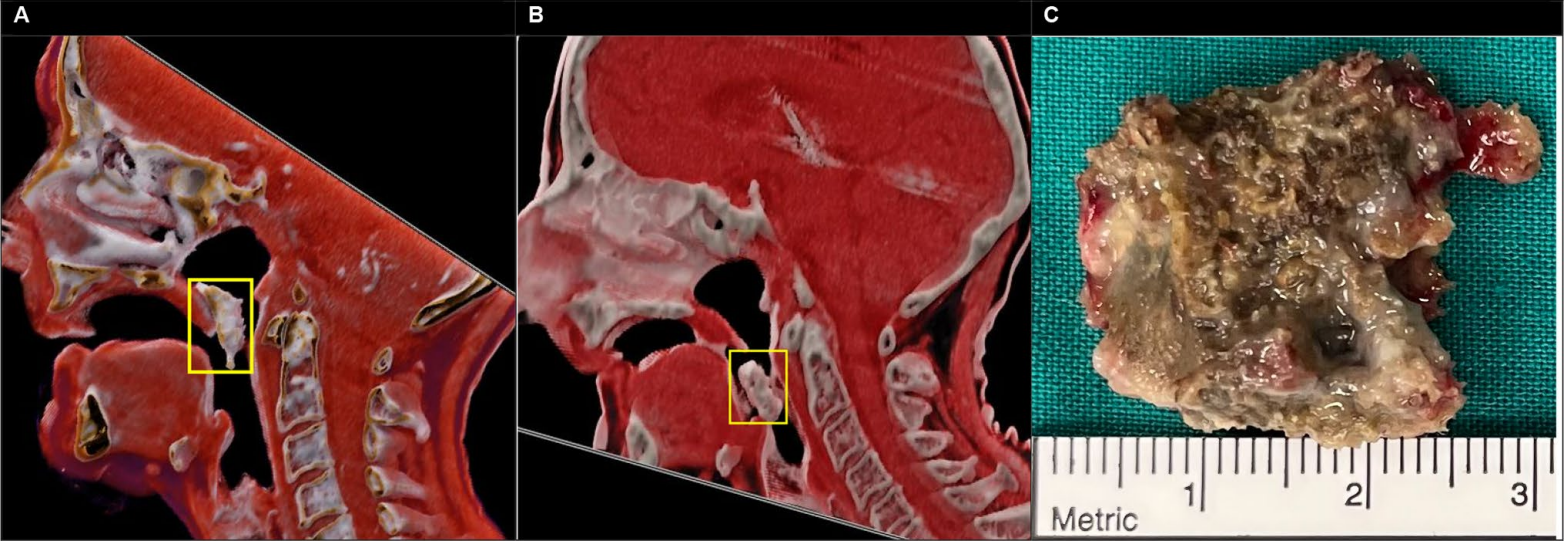
VRT-reconstruction of the CT scans (A on March 10th 2023 and B on March 12th 2023) showing that the dislocation of the fractured clivus caused an almost complete obstruction of the laryngeal inlet within 48 h (**A** + **B**). The final picture of the dislocated clival sequestrum after successful transoral removal (**C**)

After ruling out any possible cervical spine instability, the patient’s airway was secured with an awake tracheostomy and the clival sequestrum was removed transorally (Fig. 2C). The pathohistological workup of the 2.2 × 2.1 × 0.5 cm bone fragment revealed infected osteonecrosis with abundant actinomyces and coccoid bacterial infection. After consultation of an infectious disease specialist, the patient was put on metronidazole and ceftriaxone for only 10 days. Because of a treatment acquired Clostridium difficile colitis, the further planned long-term antibiotic treatment had to be postponed. The patient was finally discharged pain-free (NRS 0–1/10) and decannulated without CSF fistula after a total inpatient stay of 20 days. The interdisciplinary Skull Base Board further recommended a primarily conservative treatment approach with the implementation of HBO therapy.

## Discussion

The illustrated case demonstrates another rare complication of skull base ORN among numerous others. To date, 300—350 cases of skull base ORN have been described, which is much rarer than ORN of the maxilla and mandibula, with reported incidences of 8.2–40% in the past. As accelerated fractionation without dose reduction represents a primary risk factor for the establishment of ORN, the evolution and introduction of intensity-modulated radiation therapy (IMRT) has contributed to the fact that ORN has almost vanished (0- 5%). [25–28]

Noteworthy, our patient had already developed a maxillary and mandibular ORN in 2016 and the skull base ORN manifested 7 years later. Notably, only 60 Gy with concurrent chemotherapy was applied to the jaw, representing the standard dosage for primary radiochemotherapy in the head and neck. Of course, some of the above-mentioned and well-known risk factors apply to our patient and to most head and neck patients. However, our patient obviously tends to ORN development as we could not identify additional causative factors.

In contrast, a cumulative dosage of more than 80 Gy was applied to the skull base within 1 year. The application of 80 Gy and the short application period of less than 1 year are most likely responsible for the development of skull base ORN. The broad irradiation of head and neck areas with concomitant application of chemotherapy was due to former CUP therapy regimes. [23, 24]

Consequently, the patient received the standard widefield irradiation with the above-mentioned Gy in curative intent with concomitant application of cisplatin representing the former standard of care for CUP. Hypothetically, identifying a primary tumor might have helped save radiation dose in the endangered areas of the skull, which in turn might have helped prevent ORN of the skull base and the herein-reported subsequent complication.

This case report also illustrates a prime example of correct timing in head and neck surgery. Firstly, the outpatient organized CT scan was performed in June 2021, and, for whatever reason, the referring colleagues have not undertaken any further actions. Consequently, the question whether an appropriate treatment of the skull base ORN 18 months earlier would have prevented this reported complication is hypothetical. Second, the reasons for the 48 h time gap between the 10th and 12th of march resulted from a complex decision-making process. On one hand, the radiological diagnosis of this exceptional, uncommon situation required additional time, and more importantly, the neurosurgical evaluation took time to exclude cervical instability. Once more it is noteworthy to mention that the patient was permanently monitored and under surveillance and that the final dislocation of the clival sequestrum occurred within hours.

To the best of our knowledge, this is indeed the first case of skull base ORN with dislocation of the ventral part of the clivus and consecutive dislocation to the pharyngeal tube and laryngeal inlet, causing a major airway problem. Exceptional, in our opinion, is the fact that the patient had no further complaints like a CSF leak, pneumocephalus, CNS infection or any sign of vertebral instability. In the end, the patient was discharged with significant pain relief (NRS 0–1) and decannulated for further ORN-specific therapy (HBO).

## Data Availability

The data that support the findings of this study are available from the corresponding author, upon reasonable request.

## References

[1] Eiving J (1926). Radiation osteitis. Acta Radiol.

[2] Teng MS, Futran ND (2005). Osteoradionecrosis of the mandible. Curr Opin Otolaryngol Head Neck Surg.

[3] Marx RE, Johnson RP (1987). Studies in the radiobiology of osteoradionecrosis and their clinical significance. Oral Surg Oral Med Oral Pathol.

[4] Zhuang Q, Zhang Z, Fu H, He J, He Y (2011). Does radiation-induced fibrosis have an important role in pathophysiology of the osteoradionecrosis of jaw?. Med Hypotheses.

[5] Delanian S, Lefaix JL (2004). The radiation-induced fibroatrophic process: therapeutic perspective via the antioxidant pathway. Radiother Oncol.

[6] Hoff AO, Toth B, Hu M, Hortobagyi GN, Gagel RF (2011). Epidemiology and risk factors for osteonecrosis of the jaw in cancer patients. Ann N Y Acad Sci.

[7] Glanzmann C, Gratz KW (1995). Radionecrosis of the mandibula: a retrospective analysis of the incidence and risk factors. Radiother Oncol.

[8] Goldwaser BR, Chuang SK, Kaban LB, August M (2007). Risk factor assessment for the development of osteoradionecrosis. J Oral Maxillofac Surg.

[9] Nabil S, Samman N (2012). Risk factors for osteoradionecrosis after head and neck radiation: a systematic review. Oral Surg Oral Med Oral Pathol Oral Radiol.

[10] Murray CG, Daly TE, Zimmerman SO (1980). The relationship between dental disease and radiation necrosis of the mandible. Oral Surg Oral Med Oral Pathol.

[11] Reuther T, Schuster T, Mende U, Kübler A (2003). Osteoradionecrosis of the jaws as a side effect of radiotherapy of head and neck tumour patients–a report of a thirty year retrospective review. Int J Oral Maxillofac Surg.

[12] Shaikh N, Makary CA, Ryan L, Reyes C (2021). Treatment outcomes for osteoradionecrosis of the central skull base: a systematic review. J Neurol Surg B Skull Base.

[13] Delanian S, Chatel C, Porcher R, Depondt J, Lefaix JL (2011). Complete restoration of refractory mandibular osteoradionecrosis by prolonged treatment with a pentoxifylline-tocopherol-clodronate combination (PENTOCLO): a phase II trial. Int J Radiat Oncol Biol Phys.

[14] Delanian S, Depondt J, Lefaix JL (2005). Major healing of refractory mandible osteoradionecrosis after treatment combining pentoxifylline and tocopherol: a phase II trial. Head Neck.

[15] Jacobson AS, Buchbinder D, Hu K, Urken ML (2010). Paradigm shifts in the management of osteoradionecrosis of the mandible. Oral Oncol.

[16] Chronopoulos A, Zarra T, Ehrenfeld M, Otto S (2018). Osteoradionecrosis of the jaws: definition, epidemiology, staging and clinical and radiological findings. A concise review Int Dent J.

[17] Hirsch DL, Bell RB, Dierks EJ, Potter JK, Potter BE (2008). Analysis of microvascular free flaps for reconstruction of advanced mandibular osteoradionecrosis: a retrospective cohort study. J Oral Maxillofac Surg.

[18] Shaha AR, Cordeiro PG, Hidalgo DA (1997). Resection and immediate microvascular reconstruction in the management of osteoradionecrosis of the mandible. Head Neck.

[19] Huang WB, Wong STS, Chan JYW. Role of surgery in the treatment of osteoradionecrosis and its complications after radiotherapy for nasopharyngeal carcinoma. Head Neck 2018;40(02):369–376 23 10.1002/hed.24973 28990255

[20] Daoudi H, Labeyrie MA, Guillerm S (2020). Multimodal strategy for the management of sphenoid osteoradionecrosis: preliminary results. Laryngoscope Investig Otolaryngol.

[21] Brand Y, Lim E, Waran V, Prepageran N (2015). Endoscopic transpterygoidal repair of a large cranial defect with cerebrospinal fluid leak in a patient with extensive osteoradionecrosis of the skull base: case report and technical note. J Laryngol Otol.

[22] Han P, Wang X, Liang F (2018). Osteoradionecrosis of the skull base in nasopharyngeal carcinoma: incidence and risk factors. Int J Radiat Oncol Biol Phys.

[23] National Comprehensive Cancer Network. NCCN Clinical Practice Guidelines in Oncology: Head and Neck Cancers. 2016;V1.2016. https://www.nccn.org/professionals/physician_glsf_guidelines.asp.Accessed August 1 2017.

[24] Eskander A, Ghanem T, Agrawal A; Education Committee of American Head and Neck Society (AHNS). AHNS Series: Do you know your guidelines? Guideline recommendations for head and neck cancer of unknown primary site. Head Neck. 2018 Mar;40(3):614–621. 10.1002/hed.25026. Epub 2017 Nov 21. PMID: 29159978. 10.1002/hed.25026 29159978

[25] S. Nabil, N. Samman Incidence and prevention of osteoradionecrosis after dental extraction in irradiated patients: a systematic review Int J Oral Maxillofac Surg, 40 (2011), pp. 229–243, 10.1016/j.ijom.2010.10.005 10.1016/j.ijom.2010.10.005 21115324

[26] A.A. Owosho, C.J. Tsai, R.S. Lee, H. Freymiller, A. Kadempour, S. Varthis, et al.The prevalence and risk factors associated with osteoradionecrosis of the jaw in oral and oropharyngeal cancer patients treated with intensity-modulated radiation therapy (IMRT): The Memorial Sloan Kettering Cancer Center experience Oral Oncol, 64 (2017), pp. 44–51, 10.1016/j.oraloncology.2016.11.015 10.1016/j.oraloncology.2016.11.015 PMC5560021 28024723

[27] Di Maio P, Iocca O, De Virgilio A (2019). Role of palatine tonsillectomy in the diagnostic workup of head and neck squamous cell carcinoma of unknown primary origin: a systematic review and meta-analysis. Head Neck.

[28] Al-Lami A, Gao C, Saddiq M (2022). Reducing the unknowns: A systematic review & meta-analysis of the effectiveness of trans-oral surgical techniques in identifying head and neck primary cancer in carcinoma unknown primary. Oral Oncol.

